# Risk factors for worsening of somatic symptom burden in a prospective cohort during the COVID-19 pandemic

**DOI:** 10.3389/fpsyg.2022.1022203

**Published:** 2022-10-20

**Authors:** Petra Engelmann, Bernd Löwe, Thomas Theo Brehm, Angelika Weigel, Felix Ullrich, Marylyn M. Addo, Julian Schulze zur Wiesch, Ansgar W. Lohse, Anne Toussaint

**Affiliations:** ^1^Department of Psychosomatic Medicine and Psychotherapy, University Medical Center Hamburg-Eppendorf, Hamburg, Germany; ^2^Department of Internal Medicine, University Medical Center Hamburg-Eppendorf, Hamburg, Germany; ^3^German Center for Infection Research (DZIF), Partner Site Hamburg-Lübeck-Borstel-Riems, Hamburg, Germany

**Keywords:** COVID-19, Long COVID, risk factors, somatic symptom burden, persistent somatic symptoms

## Abstract

**Introduction:**

Little is known about risk factors for both Long COVID and somatic symptoms that develop in individuals without a history of COVID-19 in response to the pandemic. There is reason to assume an interplay between pathophysiological mechanisms and psychosocial factors in the etiology of symptom persistence.

**Objective:**

Therefore, this study investigates specific risk factors for somatic symptom deterioration in a cohort of German adults with and without prior SARS-CoV-2 infection.

**Methods:**

German healthcare professionals underwent SARS-CoV-2 IgG antibody testing and completed self-rating questionnaires at baseline and 21 months later between April 2020 and February 2022. Differences in variables between the time points were analyzed and a regression analysis was performed to predict somatic symptom deterioration at follow-up.

**Results:**

Seven hundred fifty-one adults completed both assessments. Until follow-up, *n* = 58 had contracted SARS-CoV-2 confirmed by serology. Between baseline and follow-up, signs of mental and physical strain increased significantly in the sample. Symptom expectations associated with COVID-19 and a self-reported history of COVID-19, but not serologically confirmed SARS-CoV-2 infection, significantly predicted somatic symptom deterioration at follow-up. A further predictor was baseline psychological symptom burden.

**Conclusions:**

This study supports a disease-overarching biopsychosocial model for the development of burdensome somatic symptoms during the COVID-19 pandemic and supports research findings that symptom burden may be more related to the psychosocial effects of the pandemic than to infection itself. Future studies on Long COVID should include SARS-CoV-2 negative control groups and consider symptom burden prior to infection in order to avoid an overestimation of prevalence rates.

## Introduction

In the third year of the global pandemic, there is growing interest in the potential long-term effects of SARS-CoV-2 infection, as studies indicate that a substantial portion of COVID-19 patients does not fully recover. Instead, ongoing symptoms like fatigue, shortness of breath, olfactory and gustatory dysfunction, and pain are reported (Lopez-Leon et al., 2021; Sudre et al., 2021; Seeßle et al., 2022). However, little is known about psychological predictors of symptom burden such as illness-related anxiety or expectations in patients with Long COVID. According to the British National Institute for Health and Care Excellence (NICE) COVID-19 guideline and the German Association of the Scientific Medical Societies (AWMF) S1-guideline “Post-COVID/Long-COVID,” this term is currently used to describe symptoms that last more than 4 weeks after the onset of a SARS-CoV-2 infection, can affect almost every system in the body, may change over time, and are not explained by an alternative diagnosis (Carfì et al., 2020; Koczulla et al., 2021; Shah et al., 2021). Also, risk factors for the development of burdensome somatic symptoms during the COVID-19 pandemic in individuals who have not been infected with SARS-CoV-2 have hardly been investigated so far.

In terms of general risk factors for the development of persistent somatic symptoms (PSS), current disease-overarching models (Henningsen et al., 2018; Löwe et al., 2022) propose a multifactorial etiology involving psychological, sociodemographic, and biomedical factors. Regarding biomedical factors, prior medical conditions (Claassen-van Dessel et al., 2018) and immunological factors (Rief and Martin, 2014) have been found to increase the predisposition for PSS, while stressors like acute infections are considered triggering factors for short-term symptoms (Löwe et al., 2016). Maintaining/aggravating factors include psychosocial factors such as anxiety (Creed, 2019; Niles and O'Donovan, 2019; Behm et al., 2021), depression (Fischer et al., 2013; Limburg et al., 2017), and psychosocial distress (Kirmayer et al., 2004; Deary et al., 2007). Beyond that, recent studies have shown that the expectation of experiencing persistent symptoms is crucial for symptom processing and persistence (Van den Bergh et al., 2017; Kube et al., 2020). Consequently, expectations, i.e., future-directed cognitions regarding the anticipated course of symptoms, have been found to predict symptom course in a wide range of medical and psychological conditions, e.g., chronic fatigue syndrome (Moss-Morris et al., 2011).

Due to the omnipresence of the topic in personal experiences and the media, the uncertainty about the course of the pandemic, the limitations regarding social exchange and activities, and other government restrictions (Holmes et al., 2020; Hossain et al., 2020), pandemic-related distress might function as a risk factor for PSS in non-infected populations. In fact, anxiety and fear of infection have been reported to occur more frequently in response to the COVID-19 outbreak compared to before the pandemic (Gallagher et al., 2020; Jungmann and Witthöft, 2020; Sauer et al., 2020; Kibbey et al., 2021). Thus, COVID-19 related health worries might promote the tendency to ascribe certain bodily perceptions and symptoms to SARS-CoV-2 infection (Taylor et al., 2020; Tull et al., 2020). The few studies investigating PSS in populations that did not contract SARS-CoV-2 confirmed somatization to be common during the pandemic (Ran et al., 2020; Hao et al., 2021) and found an association between COVID-19 related anxiety and somatic symptoms (Liu et al., 2020). Yet, studies that explicitly examine risk factors for the development of somatic symptoms in individuals without former SARS-CoV-2 infection during the pandemic are largely missing. In the analysis of an earlier follow-up time point of the here presented sample, we found baseline somatic symptom burden, higher levels of anxiety, occupation as a nurse, younger age, higher psychological symptom burden, lower efficiency, and higher fatigability at baseline to predict somatic symptom burden in a cohort without prior SARS-CoV-2 infection after 8 weeks (Engelmann et al., 2022).

Female sex, higher age, high body mass index, specific autoantibodies, viremia, and pre-existing medical conditions including type 2 diabetes can be considered biomedical risk factors for Long COVID (Townsend et al., 2020; Lopez-Leon et al., 2021; Sudre et al., 2021; Chudzik et al., 2022; Su et al., 2022). In a recent systematic review and meta-analysis of 20 studies investigating prognostic factors for Long COVID in adults previously hospitalized for COVID-19, female sex and acute disease severity emerged as independent prognostic factors for PSS at least 12 weeks after the infection (Maglietta et al., 2022).

However, current evidence shows that also a high number of COVID-19 patients without these clinical risk factors report somatic symptoms months after the initial infection (Moreno-Pérez et al., 2021). Besides, several studies indicated a small or no association between Long COVID and initial disease severity (Townsend et al., 2020; Sudre et al., 2021; Sykes et al., 2021; Bungenberg et al., 2022; Huang et al., 2022). Many symptoms of Long COVID are unspecific (Davis et al., 2021) and have been shown to frequently occur in the general population prior to the COVID-19 pandemic (Hinz et al., 2017). Therefore, it seems reasonable to assume a biopsychosocial perspective in explaining Long COVID, i.e., an interplay between pathophysiological mechanisms and psychological factors (Yelin et al., 2020; Sykes et al., 2021). Still, only a few studies have been conducted on psychological risk factors for Long COVID. Preliminary evidence points to the importance of anxiety, depression, and symptom expectations (Townsend et al., 2020; Taquet et al., 2021; Matta et al., 2022). In a recently published large cross-sectional cohort study, the self-reported belief of having been infected with SARS-CoV-2 in the past was significantly associated with somatic symptoms persisting for at least 8 weeks, while a positive serology test result was only positively associated with anosmia, with no significant interaction between self-reported COVID-19 and serology test results (Matta et al., 2022).

The congruence between risk factors described in overarching disease models for the etiology of PSS (Henningsen et al., 2018; Löwe et al., 2022) and those that have been found for Long COVID so far suggests the investigation of potential psychological predictors of worsening of somatic symptoms over the course of the pandemic in both individuals formerly infected with SARS-CoV-2 and individuals without a history of COVID-19 to be highly relevant. Most studies on Long COVID lack adequate control groups (Amin-Chowdhury and Ladhani, 2021) and simultaneous investigations of predictors of PSS in affected and unaffected populations have not yet been conducted. With regard to observation periods, the data basis of most publications refers to the first wave of infection in 2020 (Mauz et al., 2021). Follow-up assessments are necessary to identify symptom development and predictors of symptom change ever since, as the pandemic's effect may have evolved over time.

Therefore, the objective of the present study was to prospectively examine the course of somatic and psychological symptoms as well as specific risk factors for somatic symptom deterioration after 21 months in individuals with and without SARS-CoV-2 infection since baseline. To further assess the role of SARS-CoV-2 infection for self-reported somatic and psychological symptoms, we aimed to compare participants with SARS-CoV-2 positive and negative IgG antibody test results and positive and negative self-reported SARS-CoV-2 infection. In general, it was our aim to improve the understanding of some of the various hypothesized biopsychosocial risk factors involved in the development of burdensome somatic symptoms in response to the COVID-19 pandemic.

## Materials and methods

### Participants and study design

The current study used the first and last of four waves of data collection within a prospective cohort study to determine the seroprevalence of SARS-CoV-2 and to track bothersome somatic symptoms during the pandemic (Brehm et al., 2021; Engelmann et al., 2022). Data were collected among healthcare professionals working at the University Medical Center Hamburg, Germany. Inclusion criteria were at least 18 years of age, employment at the University Medical Center Hamburg, informed consent, and the ability to understand German. Recruitment was carried out by informing employees both in person and *via* an internal email newsletter. Prior to recruitment, written informed consent was obtained from all study participants. To promote participant retention, participation reminders were sent out before each data collection point via an email reminder as well as regularly via email newsletter. The study protocol was approved by the ethics board of the Hamburg Medical Chamber, Germany (PV 7298).

At baseline (T0), age, gender, and profession were assessed in a sociodemographic questionnaire. At baseline as well as at follow-up 21 months later (T1), participants completed a battery of self-rating questionnaires. Baseline data collection started at the end of April and was completed at the beginning of July 2020. Follow-up data collection took place between January and February 2022. The data collection period thus covered the peak phase of the pandemic in Germany to date. All participants underwent SARS-CoV-2 IgG antibody testing: Blood samples were taken at baseline and follow-up and a semi-quantitative SARS-CoV-2 immunoglobin (Ig) G enzyme-linked immunosorbent assay (ELISA) targeting the S1-Domain of the S-protein spike protein subunit (Euroimmun Medizinische Labordiagnostika, Lübeck, Germany) was performed according to the manufacturer's instructions. A stringent cut-off ratio of the extinction of the serum sample over the extinction of the calibrator of ≥ 1.5 for positive results was used, which has been shown to display a specificity of 100% (Pflüger et al., 2020), in order to account for the low prevalence environment.

### Instruments

For a detailed description of the instruments used, see Engelmann et al. (2022). In short, somatic symptom burden was assessed *via* the Somatic Symptom Scale-8 [SSS-8 (Gierk et al., 2014)] which measures eight common somatic symptoms (e.g., shortness of breath and joint pain) on a five-point response range (0–4). Sum scores can be categorized into minimal (0–3 points), low (4–7 points), medium (8–11 points), high (12–15 points), or very high (16–32 points) somatic symptom burden. Psychological symptom burden was inquired via the Somatic Symptom Disorder–B Criteria Scale [SSD-12 (Toussaint et al., 2016)]. Consisting of 12 items, it uses four items respectively to ask about psychological burden related to the somatic symptoms on an emotional, cognitive, and behavioral level. A score between 0 and 4 is used for each of the 12 items and a total score is calculated from the sum of the items. Symptom expectations associated with COVID-19 were assessed using a self-developed numeric rating scale (NRS: “How much do you expect to be burdened by symptoms in case of a COVID-19 infection?”) with a range from 0 to 10. Participants were instructed to answer the item on symptom expectations only if they had not been infected with SARS-CoV-2 yet. Depression and anxiety severity were examined with the PHQ-4 [Patient Health Questionnaire (Kroenke et al., 2009)], the ultrashort form of the Patient Health Questionnaire (PHQ–D) which consists of 4 items. We used its depression (PHQ-2) and anxiety subscales (GAD-2) where responses are scored between 0 and 3, resulting in a total score between 0 and 6. To assess the internal consistency of the used scales in our sample, Cronbach's alpha was calculated. All reliabilities were acceptable to excellent (SSS-8: 0.79, SSD-12: 0.93, PHQ-4: 0.84).

Additionally, participants were asked by a dichotomous item if they had a history of COVID-19 (“I have been diagnosed with SARS-CoV-2 infection by nasal/pharyngeal swabbing”), and if they suffer or have suffered from Long COVID (“Do you suffer or have you suffered from Long COVID syndrome?”) with three response options (“No”; “Yes, but now I have overcome Long COVID syndrome”; “Yes, I am still suffering from Long COVID syndrome”). Participants were also asked if and how often they had been vaccinated against COVID-19. Except for the items on Long COVID and vaccination, all instruments were employed at both baseline and follow-up assessment.

### Statistical analyses

Statistical analyses were conducted using IBM SPSS 27. For sample characteristics, descriptive statistics were used. In order to detect potential selection bias, dropout analyses were performed. Differences between psychological variables at T0 and T1 within the study group were compared with paired samples *t*-tests.

To explore the impact of non-modifiable and modifiable factors on somatic symptom change between T0 and T1, a multiple linear regression analysis controlling for age, gender, and somatic symptom burden at T0 was conducted. As pre-specified predictors, the regression model included SARS-CoV-2 infection since T0 determined by IgG antibody test and self-reported SARS-CoV-2 infection since T0, as well as modifiable explanatory baseline measures (psychological symptom burden, symptom expectations associated with COVID-19, depression severity, and anxiety severity). The dependent variable was somatic symptom change (Δ SSS-8) between T0 and T1. Multicollinearity was examined through tolerance and variance inflation factor criteria. The assumption was not violated.

Comparisons between participants differing in terms of SARS-CoV-2 IgG antibody test result at T1 and self-reported belief of SARS-CoV-2 infection since T0 on the continuous study variables at T1 were computed by forming four groups (“serology and belief no,” “serology and belief yes,” “serology no and belief yes,” “serology yes and belief no”) and performing one-way ANOVA.

## Results

### Sample characteristics

In total, *N* = 1,792 healthcare professionals aged ≥ 18 years (27.9% nurses, 20.5% medical doctors, 9.4% medical (technical) assistants, 6.3% scientists, 5.6% administrative employees, 20.8% others such as pharmacists, psychologists, social educations workers, students) were recruited. Of the 1,792 participants at baseline, *n* = 751 (41.9%) completed the measures at follow-up after 21 months. The mean follow-up interval was *M* = 20.07 (*SD* = 0.37) months. Compared to the dropout group, the study sample was characterized by significantly older age (study group: *M* = 40.32, *SD* = 11.79; dropout group: *M* = 35.58, *SD* = 11.32; *p* < 0.001), a higher percentage of females (study group: 77.9% females, dropout group: 65.0% females; *p* < 0.001), and a significantly lower depression severity (study group: *M* = 0.88, *SD* = 1.04; dropout group: *M* = 1.02, *SD* = 1.11; *p* = 0.01). All other scores were within similar range in both groups. The study sample had a mean age of *M* = 40.26 years (*SD* = 11.75). At follow-up, 93.3% of the study sample reported to be fully vaccinated against COVID-19, i.e., three vaccinations, according to the recommendation at that time.

### Comparison between study variables at baseline and follow-up

Table 1 shows the comparison between relevant variables within the study sample at baseline and follow-up. Significant differences were found between the two time points on all variables, with significantly higher scores of somatic and psychological symptom burden as well as depression and anxiety severity at follow-up. Symptom expectations associated with COVID-19 significantly decreased over time. According to the effect sizes, all differences are of small magnitude (Cohen, 2013).

**Table 1:** Comparison between study variables at T0 and T1 (*n* = 751).

**Variable**	**T0**	**T1**	***t*; *p*-Value**	**ES**
Somatic symptom burden (SSS-8), *M (SD)*	3.15 (3.41)	4.89 (4.21)	*t* (750) = −12.68; ***p*** **<** **0.001**	−0.46
Psychological symptom burden (SSD-12), *M (SD)*	5.28 (5.73)	7.38 (7.52)	*t* (750) = −10.34; ***p*** **<** **0.001**	−0.38
Symptom expectations (NRS), *M (SD)*	4.03 (2.07)	3.58 (1.92)	*t* (750) = 5.07; ***p*** **<** **0.001**	0.20
Depression severity (PHQ-2), *M (SD)*	0.88 (1.04)	1.14 (1.17)	*t* (750) = −6.03; ***p*** **<** **0.001**	−0.22
Anxiety severity (GAD-2), *M (SD)*	0.72 (1.03)	0.96 (1.23)	*t* (750) = −5.50; ***p*** **<** **0.001**	−0.20

M, mean; SD, standard deviation; ES, effect size (Cohen's d); SSS-8, Somatic Symptom Scale-8 (range: 0-32); SSD-12, Somatic Symptom Disorder–B Criteria Scale (range: 0–48); NRS, numeric rating scale, symptom expectations: “How much do you expect to be burdened by symptoms in case of a COVID-19 infection?” (range: 0–10); PHQ-2, Patient Health Questionnaire, 2-Item depression scale (range: 0–12); GAD-2, Generalized Anxiety Disorder Scale, 2-item anxiety scale (range: 0–12); significant p-values printed in bold mark.

### Prediction of somatic symptom deterioration

Between baseline and follow-up, participants' somatic symptom burden increased by an average of 1.74 points on the SSS-8 (range from worsening by 18 points to improvement by 11 points, *SD* = 3.76). Predictors of somatic symptom deterioration between baseline and follow-up are shown in Table 2. According to our regression model, 17% of the variance in somatic symptom change could be explained by three of the included factors. The strongest predictor of somatic symptom deterioration after 21 months was psychological symptom burden at baseline. In addition, participants with higher symptom expectations associated with COVID-19 at baseline as well as those who reported a SARS-CoV-2 infection since baseline were more likely to report somatic symptom deterioration after 21 months.

**Table 2:** Multiple linear regression analysis to test predictors of somatic symptom change (Δ SSS-8) at follow-up, adjusting for age, gender, and somatic symptom burden at baseline (*n* = 751).

**Predictor**	***b (SE)***	**ß**	***p***
SARS-CoV-2 infection since baseline determined by IgG antibody test	−1.27 (0.77)	−0.09	0.100
Self-reported SARS-CoV-2 infection since baseline	1.86 (0.78)	0.13	**0.017**
Psychological symptom burden at baseline (SSD-12)	0.11 (0.03)	0.16	**< 0.001**
Symptom expectations at baseline (NRS)	0.20 (0.07)	0.11	**0.004**
Depression severity at baseline (PHQ-2)	0.21 (0.16)	0.06	0.197
Anxiety severity at baseline (GAD-2)	0.14 (0.17)	0.04	0.410

SSS-8, Somatic Symptom Scale-8; SSD-12, Somatic Symptom Disorder–B Criteria Scale; NRS, numeric rating scale, symptom expectations: “How much do you expect to be burdened by symptoms in case of a COVID-19 infection?” (range: 0–10); PHQ-2, Patient Health Questionnaire, 2-Item depression scale (range: 0–12); GAD-2, Generalized Anxiety Disorder Scale, 2-item anxiety scale (range: 0–12); adjusted R^2^ = 0.167, Δ R^2^ = 0.178, Δ F = 15.618, df = (9, 649), sig. ΔF = < 0.001; significant p-values printed in bold mark.

Participants' serologically confirmed SARS-CoV-2 infection since baseline determined by IgG antibody test, as well as depression severity and anxiety severity at baseline did not significantly contribute to the explained variance in somatic symptom deterioration at follow-up.

### Group comparisons on study variables

At baseline, none of the participants of our study sample had a history of COVID-19 according to both SARS-CoV-2 IgG antibody test result as well as self-report. During the 2 years of the study period, *n* = 58 participants had contracted SARS-CoV-2 confirmed by positive SARS-CoV-2 IgG antibody test result, while at follow-up *n* = 68 participants reported to have been through a SARS-CoV-2 infection. Of those who reported a former SARS-CoV-2 infection, in *n* = 11 this could not be confirmed by a positive antibody test result. Of those participants with a positive antibody test result, *n* = 13 reported no history of COVID-19, i.e., had suffered from a hidden infection.

Comparisons between the four groups differing in terms of SARS-CoV-2 IgG antibody test result at follow-up and self-reported belief of SARS-CoV-2 infection since baseline (“serology and belief no,” “serology and belief yes,” “serology no and belief yes,” “serology yes and belief no”) on the study variables somatic and psychological symptom burden, symptom expectations, and depression and anxiety severity at follow-up are shown in Table 3. One-way ANOVA yielded no significant differences between groups on any of the variables. Regarding the SSS-8 scores, there was a trend toward a significant between-group difference (*p* = 0.060). The “serology no and belief yes” group stated the highest SSS-8 mean score of all of the four groups at follow-up. The second highest SSS-8 score was reported by the “serology yes and belief yes” group, followed by the “serology no and belief no” group. The “serology yes and belief no” group stated the lowest mean somatic symptom burden.

**Table 3:** Comparison between groups differing in terms of SARS-CoV-2 serology and self-reported belief of SARS-CoV-2 infection on study variables at T1 (*n* = 684).

**Variable**	**Serology**–		**Serology**+			
	**Belief– (*n* = 615)**	**Belief+ (*n* = 11)**	**Belief– (*n* = 13)**	**Belief+ (*n* = 45)**	***F*(4, 746)**	**η2**
Somatic symptom burden (SSS-8), *M (SD)*	4.82 (4.12)	7.55 (4.06)	2.85 (3.34)	5.64 (4.88)	2.27	0.01
Psychological symptom burden (SSD-12), *M (SD)*	7.35 (7.34)	10.73 (10.57)	6.15 (8.36)	7.27 (7.52)	0.64	0.00
Symptom expectations (NRS), *M (SD)*	3.56 (1.93)	–	3.42 (1.44)	–	0.47	0.00
Depression severity (PHQ-2), *M (SD)*	1.15 (1.16)	1.18 (1.78)	1.23 (0.93)	0.96 (1.21)	0.32	0.00
Anxiety severity (GAD-2), *M (SD)*	0.94 (1.19)	1.55 (1.75)	0.77 (1.24)	0.82 (1.19)	1.28	0.01

Serology test result or self-reported belief of SARS-CoV-2 infection negative (–) or positive (+); M, mean; SD, standard deviation; SSS-8, Somatic Symptom Scale-8 (range: 0–32); SSD-12, Somatic Symptom Disorder–B Criteria Scale (range: 0–48); NRS, numeric rating scale, symptom expectations: “How much do you expect to be burdened by symptoms in case of a COVID-19 infection?” (range: 0–10); PHQ-2, Patient Health Questionnaire, 2-Item depression scale (range: 0–12); GAD-2, Generalized Anxiety Disorder Scale, 2-item anxiety scale (range: 0–12); missing values on symptom expectations are due to the fact that participants were instructed to answer the item only if they had not been infected with SARS-CoV-2 yet; all p-values > 0.05.

In terms of Long COVID, *n* = 13 of the *n* = 58 participants with a prior infection confirmed by positive SARS-CoV-2 IgG antibody test result stated at follow-up to suffer (*n* = 7) or to have suffered (*n* = 6) from Long COVID. Beyond that, *n* = 3 participants without a positive antibody test result reported to have experienced Long COVID in the past. Due to the small sample size of this group, we did not perform any further statistical analyses regarding Long COVID.

## Discussion

### Risk factors for somatic symptom deterioration during the COVID-19 pandemic

The results of this prospective cohort study indicate symptom expectations, as opposed to serologically confirmed SARS-CoV-2 infection, to be relevant for the report of worsening of somatic symptoms over the course of the pandemic. Symptom expectations as well as a self-reported SARS-CoV-2 infection since baseline were found to be significant predictors of somatic symptom deterioration after 21 months, while actual infection confirmed by reliable SARS-CoV-2 IgG antibody testing was not. Baseline psychological burden associated with somatic symptoms emerged as a further predictor of somatic symptom deterioration at follow-up. We found an interesting, yet not significant trend in our data, revealing that individuals who had a negative SARS-CoV-2 IgG antibody test but who believed they have been infected since baseline showed higher somatic symptom burden after 21 months than individuals with a positive SARS-CoV-2 IgG antibody test who did not know about their infection. This result needs confirmation in well-powered samples, however, the results of this study support the importance of disease-overarching biopsychosocial models for the development of PSS (Henningsen et al., 2018; Löwe et al., 2022) which might also be relevant for symptom persistence in Long COVID.

Our findings are in line with a cross-sectional population-based French cohort study of *N* = 26,823 participants in which the self-reported belief of having been infected with SARS-CoV-2 in the past was significantly associated with symptom persistence of 8 weeks or more. Whereas, a positive serology test result was only positively associated with the symptom of anosmia, with no significant interaction between self-reported COVID-19 and serology test results (Matta et al., 2022). The present study not only supports these findings, but our prospective design provides additional corroboration of the importance of expectations for the development of bothersome somatic symptoms in response to the COVID-19 pandemic regardless of SARS-CoV-2 serology status. A missing link between self-reported symptoms and biological abnormalities has also recently been reported in a patient group with acute SARS-CoV-2 infection. In an observational cohort study of non-hospitalized adolescents and young adults involving *n* = 405 positive COVID-19 cases and *n* = 111 non-COVID-19 controls, Lund Berven et al. (2022) found a higher incidence of COVID-typical clinical symptoms among the COVID-19 cases. However, clinical symptoms were independent of objective inflammatory and pulmonary function markers. The power of expectations to predict symptom course, treatment benefit, and negative treatment side effects has been confirmed before the pandemic for a variety of medical illnesses and also for somatic symptoms relevant in Long COVID, such as pain and fatigue (Goffaux et al., 2009; Moss-Morris et al., 2011; Vase et al., 2015; Schmitz et al., 2019).

Our results are consistent with previous studies in which prior psychological symptom burden has been found to be a risk factor for PSS (Voigt et al., 2013; Klaus et al., 2015; Löwe et al., 2021). In the analysis of an earlier follow-up time point of the here presented sample, baseline psychological symptom burden was found to be a predictor of somatic symptom burden in a cohort without prior SARS-CoV-2 infection after 8 weeks (Engelmann et al., 2022). In contrast to the previous analysis, baseline anxiety did not predict somatic symptom deterioration at the 21 months follow-up, while symptom expectations did. Anxiety seems to be an important predictor of somatic symptom burden in the short term, whereas symptom expectations seem to be more relevant in the longer term. This finding may be influenced by a measurement bias. While we measured anxiety with the GAD-7, assessing general anxiety, we inquired symptom expectations specifically addressing COVID-19. Accordingly, the directly COVID-19 related question appears to better predict somatic symptom burden at longer-term follow-up than general questions on anxiety and worrying.

### Development of psychological distress over time

Over the period of data collection from spring 2020 until the beginning of 2022, both psychological and somatic symptom burden increased significantly in our sample. Publications of data collected early in the pandemic reported deleterious effects on mental health with significantly higher levels of depression and anxiety than pre-pandemic estimates in the general population (Ettman et al., 2020; Pierce et al., 2020; Nochaiwong et al., 2021; Blasco-Belled et al., 2022) and in samples of healthcare professionals (Pappa et al., 2020; Bekele and Hajure, 2021; Hao et al., 2021; Blasco-Belled et al., 2022). In contrast, some later studies suggest that after an initial peak, psychological distress gradually declined over time until almost returning to baseline levels by mid-2020 (Fancourt et al., 2021; Aknin et al., 2022; Robinson et al., 2022). The latter results cannot be confirmed by our data. However, it is to say that even at follow-up, overall somatic and psychological impairment in our study must be considered low to moderate compared to normative values (Kroenke et al., 2009; Gierk et al., 2014; Toussaint et al., 2020). Even though we did not assess symptoms before the COVID-19 outbreak in our sample, this seems to be in line with studies of representative German samples that found only small increases of symptoms of depression and anxiety during the COVID-19 pandemic in comparison to pre-pandemic levels (Entringer et al., 2020; Peters et al., 2020; Beutel et al., 2021). Recent findings suggest that vaccination against COVID-19 may have a positive impact on mental wellbeing (Babicki et al., 2021). Since almost our entire sample reported to be fully vaccinated, this could also play a role in our results.

### Strengths and limitations

A particular strength of this study is its prospective design, which adds further evidence to previous cross-sectional results (Matta et al., 2022), as well as the large sample of German adults which included both participants who did and who did not contract SARS-CoV-2. Most studies on Long COVID lack adequate control groups and do not consider symptom burden prior to infection, which calls published prevalences into question (Lopez-Leon et al., 2021; Ballering et al., 2022; Seeßle et al., 2022). Therefore, it was our aim to assess somatic symptom deterioration in both individuals with and without former SARS-CoV-2 infection. Moreover, there is a lack of knowledge on potential psychosocial risk factors for both the development of Long COVID and of PSS in individuals without prior SARS-CoV-2 infection during the pandemic. This is one of the first studies to simultaneously investigate risk factors for somatic symptom deterioration in a sample of affected and unaffected individuals. In contrast to most other published data so far (Mauz et al., 2021; Matta et al., 2022), our prospective study reports data taking into account almost 2 years of the pandemic event.

The small proportion of our sample of 7.7% who has been infected with SARS-CoV-2 must be considered a limitation to our study. Consequently, our comparisons between groups of SARS-CoV-2 IgG antibody test result and self-reported belief of SARS-CoV-2 infection are only interpretable to a limited extent. The percentage of previously infected individuals in our study is comparable to other reports on healthcare professionals. A systematic review and meta-analysis of infection prevalence rates in healthcare professionals across 97 healthcare settings in Europe, the United States, and Asia found the rate to be 7% based on antibody testing and 11% using reverse transcription PCR assays (Gómez-Ochoa et al., 2021). In a longitudinal study of *N* = 1,506 healthcare professionals at a German General Hospital, 165 (10.6%) participants tested positive for SARS-CoV-2 infection within a one-year period between April 2020 and April 2021 (Platten et al., 2022). Further studies with a higher number of formerly infected participants would be needed in order to confirm our results. Also, since healthcare professionals represent a high-risk group for experiencing mental health issues (Maben and Bridges, 2020) and somatic symptoms like fatigue (Kawano, 2008), our study sample could limit the generalizability of our results. The discrepancy between participants in our sample with a history of COVID-19 confirmed by positive SARS-CoV-2 IgG antibody test result and those who self-reported to have previously been diagnosed with SARS-CoV-2 infection by nasal/pharyngeal swabbing cannot be fully reconstructed on the basis of our data. The swab results themselves may have been incorrect (false positive) or participants agreed to the item because they were actually convinced of an infection even without a positive test result. Another explanation could be the antibody test results. We determined prior SARS-CoV-2 infection by SARS-CoV-2 IgG ELISA. While antibody responses have been shown to maintain for more than 1 year post infection in symptomatic patients (Scheiblauer et al., 2022), serological responses in individuals with asymptomatic or mild infections are less well understood and may decline more rapidly (Efrati et al., 2021; Tian et al., 2021). Therefore, it is possible that not all SARS-CoV-2 infections in our sample have been detected by antibody testing.

We assessed somatic symptom burden at baseline and follow-up using the SSS-8, which uses a 7-day period. Accordingly, we only measured short-term symptoms at both time points and therefore, strictly speaking, cannot draw any conclusions on persistent symptoms. We did not use a Long COVID specific instrument since harmonized core outcome sets for Long COVID conditions are currently still being developed (Munblit et al., 2022). Another limitation is that we did not examine some of the previously reported risk factors for Long COVID like body mass index, autoantibodies, or pre-existing medical or psychiatric conditions (Lopez-Leon et al., 2021; Sudre et al., 2021; Su et al., 2022), and therefore do not know about their potential relevance for our sample. Future studies should take these factors into account.

### Conclusion

The findings of this study provide evidence for the relevance of biopsychosocial risk factors (Henningsen et al., 2018; Löwe et al., 2022) in explaining both burdensome somatic symptoms in individuals formerly infected with SARS-CoV-2 as well as somatic symptoms that develop in response to the COVID-19 pandemic in individuals that have not been infected with SARS-CoV-2. Thus, our results are in line with current evidence which confirms that a high number of low-risk COVID-19 patients develop Long COVID (Moreno-Pérez et al., 2021) and shows little or no association between Long COVID and initial disease severity (Townsend et al., 2020; Sudre et al., 2021; Sykes et al., 2021; Huang et al., 2022). In particular, dysfunctional symptom expectations seem to play a major role for the report of somatic symptom deterioration in individuals who did and did not contract SARS-CoV-2, which indicates that symptoms that are actually associated with distress caused by the pandemic might be falsely attributed to effects of SARS-CoV-2 infection. Therefore, future studies on Long COVID should include control groups without former SARS-CoV-2 infection and consider symptom burden prior to infection in order to avoid an overestimation of the prevalence of Long COVID (Amin-Chowdhury and Ladhani, 2021; Ballering et al., 2022). This is of particular importance as media reports of excessive strains through the disease might provoke dysfunctional expectations and thereby contribute to a worsening of symptoms in those affected. In these studies, further psychological factors like catastrophizing, somatosensory amplification, and learning processes like avoidance behavior should be investigated to further expand our knowledge of biopsychosocial mechanisms involved in somatic symptom burden due to the COVID-19 pandemic.

Since dysfunctional symptom expectations seem be involved in the development of PSS during the COVID-19 pandemic, they should be addressed in corresponding interventions. Targeted expectation management has already been shown to improve clinical outcomes in several other medical conditions (Rief et al., 2017; Kube et al., 2018; Pan et al., 2020). According to our results, interventions should be made accessible especially for individuals with a history of symptom burden. Interventions trying to foster healthcare professionals' coping with infectious disease outbreaks mostly do not address somatic symptoms at all (Zaçe et al., 2021). With regard to future pandemics, it is important for healthcare organizations to preventively support healthcare professionals in dealing with burdensome somatic symptoms.

## Data availability statement

All data generated or analyzed during this study are included in this article. Further enquiries can be directed to the corresponding author.

## Ethics statement

The study protocol was reviewed and approved by the ethics board of the Hamburg Medical Chamber, Germany (approval number PV 7298). Written informed consent was obtained from all participants to participate in the study.

## Author contributions

TB, MA, JS, and AL developed the concept and design of the study. FU was responsible for conducting the study. PE analyzed the data and wrote the draft of this manuscript. AT provided revisions. BL and AW contributed further refinements. All authors approved the final version of the manuscript.

## Conflict of interest

The authors declare that the research was conducted in the absence of any commercial or financial relationships that could be construed as a potential conflict of interest.

## Publisher's note

All claims expressed in this article are solely those of the authors and do not necessarily represent those of their affiliated organizations, or those of the publisher, the editors and the reviewers. Any product that may be evaluated in this article, or claim that may be made by its manufacturer, is not guaranteed or endorsed by the publisher.
